# Functional connectivity of the right inferior frontal gyrus and orbitofrontal cortex in depression

**DOI:** 10.1093/scan/nsaa014

**Published:** 2020-01-28

**Authors:** Edmund T Rolls, Wei Cheng, Jingnan Du, Dongtao Wei, Jiang Qiu, Dan Dai, Qunjie Zhou, Peng Xie, Jianfeng Feng

**Affiliations:** 1 Institute of Science and Technology for Brain-inspired Intelligence, Fudan University, 200433, Shanghai, China; 2 Department of Computer Science, University of Warwick, CV4 7AL, Coventry, UK; 3 Oxford Centre for Computational Neuroscience, Oxford, UK; 4 Department of Psychology, Southwest University, Chongqing, China; 5 Key Laboratory of Cognition and Personality (SWU), Ministry of Education, Chongqing, China; 6 Institute of Neuroscience, Chongqing Medical University, Chongqing, China; 7 Chongqing Key Laboratory of Neurobiology, Chongqing, China; 8 Department of Neurology, Yongchuan Hospital of Chongqing Medical University, 402160, Chongqing, China; 9 School of Mathematical Sciences, School of Life Science and the Collaborative Innovation Center for Brain Science, Fudan University, 200433, Shanghai, China

**Keywords:** depression, orbitofrontal cortex, inferior frontal gyrus, functional connectivity, inhibition, reward, non-reward, impulsivity

## Abstract

The orbitofrontal cortex extends into the laterally adjacent inferior frontal gyrus. We analyzed how voxel-level functional connectivity of the inferior frontal gyrus and orbitofrontal cortex is related to depression in 282 people with major depressive disorder (125 were unmedicated) and 254 controls, using FDR correction *P* < 0.05 for pairs of voxels. In the unmedicated group, higher functional connectivity was found of the right inferior frontal gyrus with voxels in the lateral and medial orbitofrontal cortex, cingulate cortex, temporal lobe, angular gyrus, precuneus, hippocampus and frontal gyri. In medicated patients, these functional connectivities were lower and toward those in controls. Functional connectivities between the lateral orbitofrontal cortex and the precuneus, posterior cingulate cortex, inferior frontal gyrus, ventromedial prefrontal cortex and the angular and middle frontal gyri were higher in unmedicated patients, and closer to controls in medicated patients. Medial orbitofrontal cortex voxels had lower functional connectivity with temporal cortex areas, the parahippocampal gyrus and fusiform gyrus, and medication did not result in these being closer to controls. These findings are consistent with the hypothesis that the orbitofrontal cortex is involved in depression, and can influence mood and behavior via the right inferior frontal gyrus, which projects to premotor cortical areas.

## Introduction

Resting state functional connectivity between brain areas, which reflects correlations of activity, is a fundamental tool in helping to understand the brain regions with altered connectivity and function in mental disorders (Deco and Kringelbach, 2014), and differences in functional connectivity in depression have been described in a number of brain areas, including the default mode network, a fronto-striatal network, a fronto-parietal network, a ‘limbic’ network, the subgenual cingulate cortex and the amygdala (Connolly *et al.,* 2013; Kaiser *et al.,* 2015; Drysdale *et al.,* 2017; Helm *et al.,* 2018; Cheng *et al.,* 2018a; Rolls *et al.,* 2019a). However, there is considerable evidence that the orbitofrontal cortex is a key brain region involved in emotion, and it therefore is a strong candidate brain region for a role in depression (Rolls, 2014, 2017, 2018a,b,c). This hypothesis has been followed up, and there is now evidence for differences in orbitofrontal cortex functional connectivity in depression (Cheng *et al.,* 2016, 2018a,b,c; Rolls *et al.,* 2019a). However, in a number of these investigations of the orbitofrontal cortex, differences of functional connectivity in depression have also been found with the laterally adjacent right inferior frontal gyrus with a number of brain regions, including the precuneus (Cheng *et al.,* 2018c), posterior cingulate cortex (Cheng *et al.,* 2018b) and amygdala (Cheng *et al.,* 2018a). Indeed, what is cytoarchitecturally the lateral orbitofrontal cortex BA 47/12 extends round the inferior frontal convexity into what topologically is the ventral part of the inferior frontal gyrus (Öngür *et al.,* 2003; Price, 2006; Saleem *et al.,* 2014). Moreover, a parcellation study of the orbitofrontal cortex, anterior cingulate cortex and inferior frontal gyrus has shown that the orbital part of the inferior frontal gyrus has similar functional connectivity with other brain regions as the lateral orbitofrontal cortex (Du *et al.,* 2020). [In the automated anatomical labeling atlases 2 and 3 (AAL2 and AAL3) (Rolls *et al.,* 2015, 2020), this brain region is termed Frontal_Inf_Orb or IFGorb, as shown in Supplementary Table S3]. In this context, we therefore wished to analyze at the voxel level exactly which parts of the orbitofrontal cortex and the laterally adjacent inferior frontal gyrus have altered functional connectivity in depression.

The aim of the present paper was therefore to examine the functional connectivity of the orbitofrontal cortex and inferior frontal gyrus in depression at the voxel level. We analyzed every orbitofrontal cortex and inferior frontal gyrus voxel for significantly different functional connectivity with voxels throughout the rest of the brain in depressed people *vs* controls, in order to advance understanding of the orbitofrontal cortex and inferior frontal gyrus and depression. The advantage of voxel-level functional connectivity is that we can show exactly which orbitofrontal cortex and inferior frontal gyrus voxels have altered functional connectivity with the exact (voxel-level) parts of other brain areas. In order to perform this voxel-level functional connectivity analysis, we utilized and required a uniquely large sample of 282 patients with major depressive disorder and 254 controls. We note that many previous studies have used relatively small sample sizes and could not analyze voxel-level functional connectivity (Helm *et al.,* 2018), and a strength of the present study is the large number of patients involved, which helps to make the findings in the present study robust, and to show the exact parts of the orbitofrontal cortex and inferior frontal gyrus and the areas to which they are connected in which voxels have different functional connectivity in depression. In this investigation, we included every part of the inferior frontal gyrus in the analysis, to provide evidence on which parts have functional connectivity that is related to depression.

Functional connectivity refers to correlations between the fMRI BOLD signals in different brain regions, and reflects direct connections between cortical areas as shown by combined anatomical pathways tracing and functional connectivity analyses in macaques, and also some trans-synaptic effects that can account for functional connectivity beyond the regions with known direct anatomical connectivity (Van Essen *et al.,* 2019). An advantage of functional connectivity is, thus, that it can reveal trans-synaptic effects, and is non-invasive and can be performed in humans. This investigation is very different from a previous analysis of functional connectivity in depression (Cheng *et al.,* 2016); in that instead of focusing on the whole brain, here, we focus on voxels in the inferior frontal gyrus and orbitofrontal cortex, and we can therefore perform a much more statistically sensitive analysis of these regions, given that the number of voxel pairs here is smaller than the 1 133 760 771 pairs across the whole brain requiring normally *P* < 10^−8^ for any voxel pair correlation difference to be significant (Cheng *et al.,* 2016). Moreover, here, we analyze the effects of the medication on the functional connectivity in depression.

A new theory of depression is that the lateral orbitofrontal cortex has an increased sensitivity of a non-reward attractor in depression, and that the reciprocally related medial orbitofrontal cortex reward system is underactive in depression (Rolls, 2016b), and there is evidence consistent with this (Eshel and Roiser, 2010; Russo and Nestler, 2013; Whitton *et al.,* 2015; Cheng *et al.,* 2016; Rolls, 2016b). It was therefore of interest in the present investigation whether the orbitofrontal cortex and inferior frontal gyrus had altered connectivity with other brain systems already implicated in depression. The new theory is that the lateral orbitofrontal frontal cortex, which is activated when expected reward is not obtained (which can cause sadness), can enter and maintain an ongoing mood state in a recurrent ‘attractor’ network more readily in depression (Rolls, 2016b, 2018a), as described more fully in the Discussion. The theory is that conversely, the medial orbitofrontal cortex, with activity related to reward, is underconnected in depression (Rolls, 2016b, 2018a, 2019b,c).

## Materials and methods

### Participants

There were 282 patients with a diagnosis of major depression, and 254 controls from Xinan (First Affiliated Hospital of Chongqing Medical School in Chongqing, China). All participants were diagnosed according to the Diagnostic and Statistical Manual of Mental Disorder-IV criteria for major depressive disorder. Depression severity and symptomatology were evaluated by the Hamilton Depression Rating Scale (HAMD, 17 items) (Hamilton, 1960) and the Beck Depression Inventory (BDI) (Beck and Beamesderfer, 1974). About 125 of the patients were not receiving medication at the time of the neuroimaging. Supplementary Table S1 provides a summary of the demographic information and the psychiatric diagnosis of the participants, and further information is provided in the Participants section of the Supplementary Material. The data set utilized here is a subset of those described in Cheng *et al.* (2016, 2018a,b) for which the present different type of analysis could be performed, and the present analysis specifically analyses the inferior frontal gyrus and orbitofrontal cortex, which was not the focus of any earlier investigation. The reason that data from only the Xinan site were used here is that these data include both medicated and non-medicated patients, and the sample size is also similar for these two groups.

### Image acquisition

Data for resting state functional connectivity analysis were collected in a 3 T MRI scanner in an 8 min period in which the participants were awake in the scanner not performing a task using standard protocols described in the Supplementary Material. Data preprocessing was standard, as has been described before (Cheng *et al.,* 2016), with details provided next.

### Image preprocessing

Data preprocessing was performed using DPARSF (Chao-Gan and Yu-Feng, 2010) (http://restfmri.net) which is a toolbox based on the SPM8 software package. The first 10 EPI scans were discarded to suppress equilibration effects. The remaining scans of each subject underwent slice timing correction by sinc interpolating volume slices, motion correction for volume-to-volume displacement, spatial normalization to standard Montreal Neurological Institute space using affine transformation and non-linear deformation with a voxel size of $3\times 3\times 3\ {\mathrm{mm}}^3$, followed by spatial smoothing (8 mm full width half maximum). To remove the sources of spurious correlations present in resting-state BOLD data, all fMRI time-series underwent bandpass temporal filtering (0.01–0.1 Hz), nuisance signal removal from the ventricles and deep white matter, and regressing out any effects of head motion using the Friston *et al*.’s 24 head motion parameters procedure (Friston *et al.,* 1996). The bandpass temporal filtering was performed after nuisance signal removal. Finally, we implemented additional careful volume censoring (‘scrubbing’) movement correction as reported by Power *et al.* (2014) to ensure that head-motion artifacts are not driving observed effects. The mean framewise displacement (FD) was computed with FD threshold for displacement being 0.5. In addition to the frame corresponding to the displaced time point, 1 preceding and 2 succeeding time points were also deleted to reduce the spill-over effect of head movements. Subjects with >10% displaced frames flagged were completely excluded from the analysis as it is likely that such high level of movement would have had an influence on several volumes. Statistical tests showed no significant differences between the groups in the six head motion parameters (three translational and three rotational direction parameters). Global signals were not regressed out, for reasons described elsewhere (Cheng *et al.,* 2016). Considering the potential effect of gender (Tomasi and Volkow, 2012), age (Geerligs *et al.,* 2015) and head motion (Power *et al.,* 2012, 2014) on functional connectivity, any effects of gender ratio, years of education, age and head motion between the patient and control groups were regressed out in all analyses. There were no differences in the gender ratios, age and mean FD (*P* > 0.05 in all cases), though the number of years of education was lower in the patients than controls.

### Statistical analysis

#### Hypothesis-based voxel-wise association studies

In the present study, each resting-state fMRI image included 47 619 voxels. For each pair of voxels, the time series were extracted and their Pearson correlation was calculated for each subject, to provide the measure of functional connectivity, followed by Fisher’s *z*-transformation. We used a linear regression model to estimate the *t* value and *P* value for the comparison between patients and controls, and this allowed covariates of no interest, namely age, gender ratio, education and head motion (mean FD), to be regressed out. This was performed on the Fisher’s *z*-transformed correlation coefficients to identify significantly altered voxel-wise functional connectivity links in patients with depression compared with controls. Given that the orbitofrontal cortex (lateral orbitofrontal cortex and medial orbitofrontal cortex) and inferior frontal gyrus (IFGtri and IFGoperc) [see Supplementary Figure S1 for the regions analyzed, and Supplementary Table S3 for the names of the automated anatomical labeling atlas 2 (Rolls *et al.,* 2015) used to name the areas in which voxels were found] had been predefined by prior hypothesis as the region of interest and had 4198 voxels, and that there were 47 619 $3\times 3\times 3\ \mathrm{mm}$ voxels in the whole automated anatomical labeling atlas (AAL2) brain (Rolls *et al.,* 2015); the number of voxel pairs in this study was approximately (4198 × 47 619), which is much smaller than the 1 133 760 771 (47 619 × 47 618/2) voxel pairs in our whole-brain study (Cheng *et al.,* 2016). This enabled significant differences in voxel-level functional connectivity of the orbitofrontal cortex and IFG with the rest of the brain to be identified in the present study. Finally, an FDR procedure was used to correct for multiple comparisons. In the present study, FDR correction for the functional connectivity between any pair of voxels was used, and results are presented based on this statistical test with FDR *P* < 0.05, corresponding to a *P* threshold of $5.45\times{10}^{-5}$ in *t*-tests. The split cross-validation analysis showed that the most significant regions were consistent across the three cases (i.e. the whole data set, the first half and the second half).

#### Visualization of the differences in functional connectivity (functional connectivity) for each voxel

To illustrate in some of the figures the extent to which voxels in different brain areas had differences of functional connectivity between patients and controls, we used a measure for the association (*MA*) between a voxel *i* and the brain disorder. This was defined as: $MA=\sum_{j=1}^{N_{\alpha}}{T}_j$, where ${N}_{\alpha}$ is the number of links between voxel $i$ and every other voxel in the brain which have a *P*-value of less than $\alpha$ (in the present study $5.45\times{10}^{-5}$) in *t*-tests comparing patients with controls; ${T}_j$ is the *t* value of the *j*’th significant link in *t*-tests comparing patients with controls. To distinguish the increased and decreased functional connectivities and avoid positive and negative *t* values canceling, we defined the *MA* of each voxel separately for increased functional connectivities and decreased functional connectivities. A larger value of $MA$ implies a more significant difference in the functional connectivity of a voxel.

## Results

### Voxel-level differences in functional connectivity in unmedicated patients with depression

All voxels with significantly different functional connectivity (FDR *P* < 0.05) between patients and controls for the medial orbitofrontal cortex, lateral orbitofrontal cortex, inferior frontal gyrus (triangular part) and inferior frontal gyrus (opercular part) are shown in Supplementary Figure S2. This figure shows the voxels within these brain regions with different functional connectivities, and the voxels in all other brain areas with which these voxels in the named brain regions have significantly different functional connectivity. In order to show which voxels in each of these other brain areas have different FC with each of the medial orbitofrontal cortex, lateral orbitofrontal cortex, inferior frontal gyrus (triangular part) and inferior frontal gyrus (opercular part), in the remainder of this section we show the results from this statistical analysis but divided separately by each region.

#### Lateral orbitofrontal cortex

**Fig. 1:**
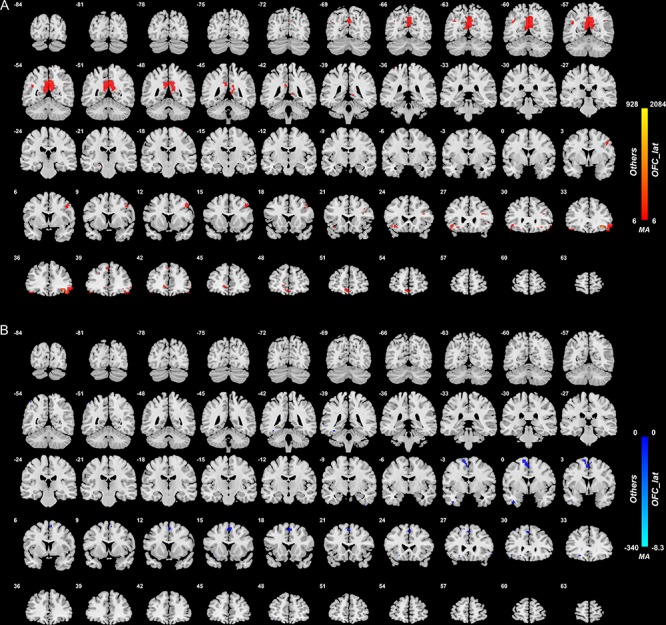
Anatomical location of voxels with significantly higher (A) and lower (B) functional connectivity with the lateral orbitofrontal cortex areas in non-medicated depression (patients—controls) obtained from the voxel-based Association Study. Blue indicates voxels with lower functional connectivity in depressed patients, and red/yellow indicates voxels with higher functional connectivity. In this and in all other figures, the level of statistical significance for the difference in functional connectivity for any voxel after correction for multiple comparisons was *P* < 0.05 FDR.

Figure 1 shows the difference in the functional connectivities related to the lateral orbitofrontal cortex in the comparison between the 125 unmedicated patients and the 254 controls (after FDR correction at *P* < 0.05). This shows that the main differences in unmedicated patients with depression are differences in functional connectivity between the lateral orbitofrontal cortex and the precuneus, posterior cingulate cortex, ventromedial prefrontal cortex (Frontal_Med_Orb in AAL2 and AAL3 (Rolls *et al.,* 2020), see Supplementary Table S2), and the angular and middle frontal gyrus, and all these functional connectivities were higher in patients with depression. No lower functional connectivity was identified except for a few voxels in the supplementary motor area. The voxels considered here were in AAL2/3 areas OFClat and IFGorb (see Supplementary Table S3). There were more voxels in the right than left lateral orbitofrontal cortex with higher functional connectivity in depression, and the middle frontal gyrus connected region was on the right.

#### Medial orbitofrontal cortex

**Fig. 2:**
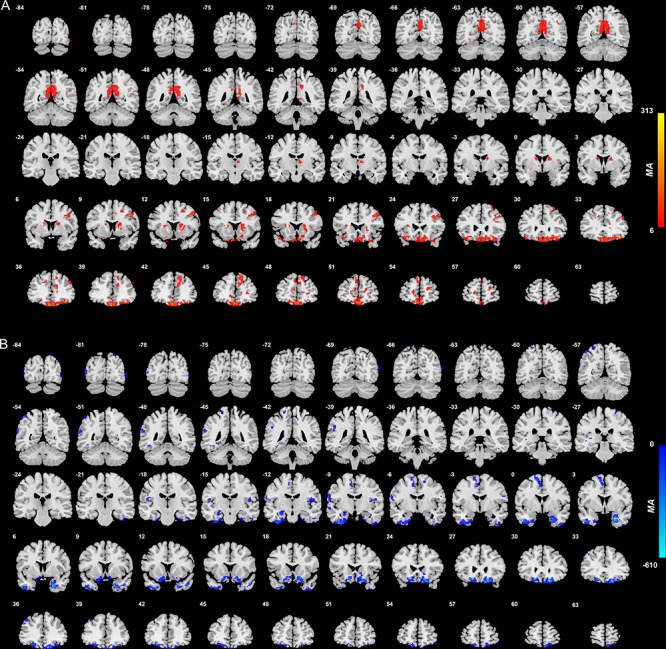
Anatomical location of voxels with significantly increased (A) and decreased (B) functional connectivity with the medial orbitofrontal cortex areas in non-medicated depression (patients—controls) obtained from the voxel-based Association Study. Blue indicates voxels with lower functional connectivity in depressed patients, and red/yellow indicates voxels with higher functional connectivity.

As shown in Figure 2 and Supplementary Table S2, there were a number of medial orbitofrontal cortex voxels with higher or lower functional connectivity with different brain areas in patients with depression compared with controls. The largest clusters of voxels with lower functional connectivity were between the medial orbitofrontal cortex and temporal cortex areas including the inferior temporal gyrus and the temporal pole (Supplementary Table S2). The other brain regions with which there was lower functional connectivity included the parahippocampal gyrus, fusiform gyrus and supplementary motor area. Also, the functional connectivities between voxels within the different AAL2 regions within the medial orbitofrontal were also lower in patients with depression. The brain areas with higher functional connectivity with voxels in the medial orbitofrontal cortex were the precuneus, right middle/superior frontal gyrus, caudate nucleus and posterior cingulate cortex (Supplementary Table S2).

#### Inferior frontal gyrus (triangular part)

**Fig. 3:**
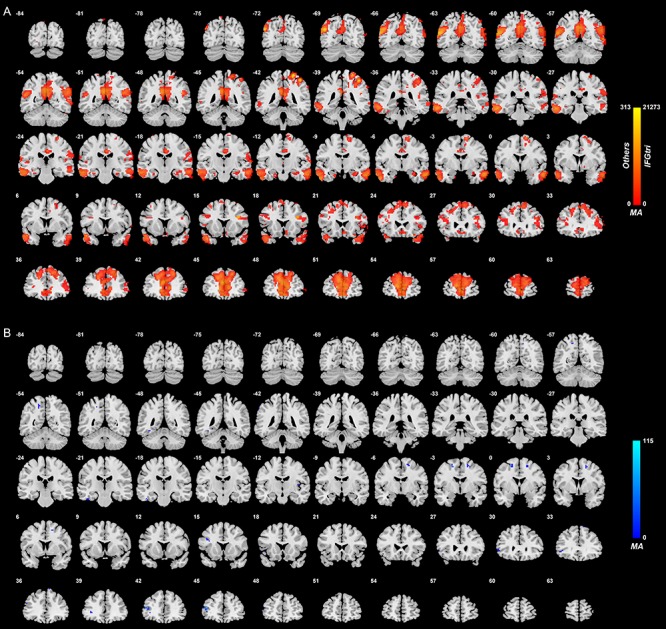
Anatomical location of voxels with significantly increased (A) and decreased (B) functional connectivity with the inferior frontal gyrus (triangular part) in non-medicated depression (patients—controls) obtained from the voxel-based Association Study. Blue indicates voxels with lower functional connectivity in depressed patients, and red/yellow indicates voxels with higher functional connectivity. The high functional connectivity with the angular gyrus is bilateral, though a little more on the right. It should be noted that some lateral orbitofrontal cortex voxels have higher functional connectivity with the inferior frontal gyrus; in that, the lateral orbitofrontal cortex BA 47/12 continues beyond the inferior convexity and up the lateral surface of the hemisphere for several mm, as shown in Figures 3 and 4 of (Öngür *et al.,* 2003).

Figure 3 shows the difference in the functional connectivities related to the inferior frontal gyrus (triangular part) in the comparison between unmedicated patients and controls (after FDR correction at *P* < 0.05). There were a number of inferior frontal gyrus (triangular part) voxels with different functional connectivity in patients with depression compared with controls. In most cases, an increase in functional connectivity was found in the group with depression. The brain areas with higher functional connectivity with voxels in the triangular part of the inferior frontal gyrus were in the prefrontal cortex including the middle and superior frontal gyrus, lateral and medial orbitofrontal cortex and cingulate cortex (Supplementary Table S2). Almost all of these differences of functional connectivity in depression involved the right inferior frontal gyrus (triangular part), as shown in Figure 3.

#### Inferior frontal gyrus (opercular part)

**Fig. 4:**
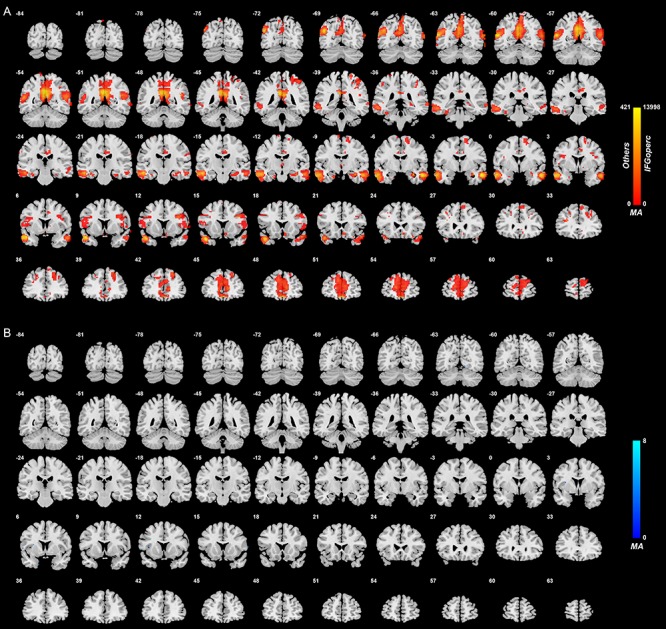
Anatomical location of voxels with significantly increased (A) and decreased (B) functional connectivity with the inferior frontal gyrus (opercular part) in non-medicated depression (patients—controls) obtained from the voxel-based Association Study. Blue indicates voxels with lower functional connectivity in depressed patients, and red/yellow indicates voxels with higher functional connectivity.

Figure 4 shows the difference in the functional connectivities related to the inferior frontal gyrus (opercular part) in the comparison between unmedicated patients and controls (after FDR correction at *P* < 0.05). There were a number of inferior frontal gyrus (opercular part) voxels with different functional connectivity in patients in the depression group compared with controls. In most cases, an increase in functional connectivity was found in the depression group. The brain areas with higher functional connectivity with voxels in the inferior frontal gyrus (opercular part) were the inferior and middle temporal gyrus and the temporal pole, the angular gyrus and the cingulate cortex, precuneus and hippocampus (Supplementary Table S2).

### Comparison of the differences in functional connectivity in unmedicated depression between the medial orbitofrontal cortex, lateral orbitofrontal cortex and inferior frontal cortex

**Fig. 5:**
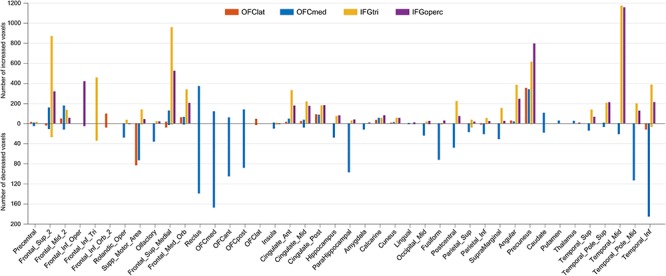
The change pattern of different brain regions. The *y*-axis indicates the number of significant voxels which show altered functional connectivity with the regions of interest (i.e. orbitofrontal cortex lat, orbitofrontal cortex med, IFGtri and IFGoperc).

Figure 5 and Supplementary Table S2 show the differences in functional connectivity between these different areas. The functional connectivities were significantly different, as shown by a Chi-Square test performed on the numbers of voxels in each area shown in Figure 5 and Supplementary Table S2 (*P* < 0.0001).

The medial orbitofrontal cortex in depression has lower functional connectivity than controls with the parahippocampal gyrus and hippocampus; temporal lobe including fusiform cortex; and to a smaller extent with the supramarginal and parietal cortex. No other of the four regions has this pattern. In addition, the medial and lateral orbitofrontal cortex have lower functional connectivity with the Supplementary Motor Area.

The lateral orbitofrontal cortex has increased functional connectivity with the precuneus—and so do all four regions. There are some voxels with higher functional connectivity with the posterior cingulate cortex, and with the angular gyrus.

The right inferior frontal gyrus pars triangularis (approximately BA45) had higher functional connectivity in the depressed group than in controls with the superior frontal gyrus (FrontalSup2 and FrontalSupMed); FrontalMedOrb (i.e. ventromedial prefrontal cortex); the anterior, middle and posterior cingulate cortex; with the left and right angular and supramarginal gyri; and with temporal cortex areas.

The inferior frontal gyrus pars opercularis (BA44) had moderately large numbers of voxels with higher functional connectivity than controls with the superior frontal gyrus (FrontalSup2 and FrontalSupMed); FrontalMedOrb (i.e. ventromedial prefrontal cortex); the anterior, middle and posterior cingulate cortex; the angular gyrus; and with temporal cortex areas. Its general pattern of functional connectivity differences is similar to the right inferior frontal gyrus pars triangularis, though with somewhat fewer voxels. (The inferior frontal gyrus pars opercularis is smaller with 699 voxels compared with the inferior frontal gyrus pars triangularis with 1447 voxels.)

### Right–left differences in functional connectivity of the lateral orbitofrontal cortex and inferior frontal gyrus in depression

It is evident in Figures 2–4 that the functional connectivity differences in the depressed group are higher in the right than left lateral orbitofrontal cortex and inferior frontal gyrus. This is emphasized for the right inferior frontal gyrus in Supplementary Figure S8, which shows many more voxels with higher functional connectivity with other brain areas in the right than left inferior frontal gyrus.

### Effects of medication

Over all regions selected for investigation, the medial and lateral orbitofrontal cortex and the inferior frontal gyrus, the functional connectivities that were higher in unmedicated patients with depression were smaller in the medicated group, and were closer to their values in the controls (Supplementary Figure S3), with the results for each region as follows.

It is clear in Supplementary Figure S4 that the functional connectivities of the lateral orbitofrontal cortex were lower in the medicated than in the unmedicated group. These effects were significant, in that voxels are only shown in this figure if *P* < 0.05 FDR corrected for the contrast medicated—non-medicated. The effect of the medication was that the functional connectivity in medicated patients was closer to that in healthy controls.

Supplementary Figure S5 shows that medial orbitofrontal cortex functional connectivities were lower in medicated than unmedicated patients. What was somewhat unexpected here was that although the medial orbitofrontal cortex has many smaller functional connectivities than in controls (Figure 2), the functional connectivities of the medial orbitofrontal cortex were even smaller in the medicated group than in the unmedicated group, though this effect was not especially clear for the medial orbitofrontal cortex with temporal lobe functional connectivities. Thus for the medial orbitofrontal cortex, the medication did not tend to bring the functional connectivities back to their value in healthy controls. This has implications for treatment that are considered in the Discussion.

Supplementary Figure S6 shows that pars triangularis of the right inferior frontal gyrus had lower functional connectivities (toward those in healthy controls) in medicated compared with unmedicated patients with major depressive disorder. These lower functional connectivities were evident with areas that included the precuneus, motor cortical areas, the temporal cortex and the supramarginal gyrus.

Supplementary Figure S7 shows that pars opercularis of the inferior frontal gyrus had lower functional connectivities (toward those in healthy controls) in medicated compared with unmedicated patients with major depressive disorder. These lower functional connectivities were evident with areas that included the cortex at the junction of the middle and inferior frontal gyri which may include high-order laryngeal cortex.

It was further of interest that although there were no significant functional connectivity differences between medicated patients and controls after voxel-level FDR correction, the functional connectivities of many voxels in the medial orbitofrontal cortex were lower (each significant at *P* < 10^−4^) in medicated patients than in controls (Supplementary Figure S9).

## Discussion

One of the very interesting new findings is on the higher functional connectivity of the inferior frontal gyrus in depression. This was with areas that included the inferior and middle temporal gyrus and the temporal pole, the angular gyrus and the cingulate cortex, precuneus and hippocampus, and the superior and middle frontal gyri. These higher functional connectivities in depression were much greater for the right than for the left inferior frontal gyri (Figures 3 and 4 and Supplementary Figure S8). Part of the interest here is that the left inferior frontal gyrus, BA45 and 44, is Broca's area and is involved in language production (Amunts *et al.,* 2010; Amunts and Zilles, 2012). In contrast, the right inferior frontal gyrus is strongly activated in the stop-signal task (Deng *et al.,* 2017), in which behavior must be checked and stopped, and damage to the right inferior frontal gyrus impairs performance on this stop-signal task (Aron *et al.,* 2014). It appears therefore that the right inferior frontal gyrus if ineffective is related to a type of impulsivity related to producing too much action, compared with being able to inhibit action (Dalley and Robbins, 2017). The suggestion that we make in accordance with the new findings presented in this investigation is that the right inferior frontal gyrus high functional connectivity in depression is related to too little tendency to initiate actions, that is, to what appears to be a lack of motivated action in depression.

Anatomical evidence is consistent with the hypothesis that the inferior frontal gyrus provides a route for the lateral orbitofrontal cortex BA 47/12 to connect to premotor areas. Area 45, the inferior frontal gyrus pars triangularis in humans, does receive from lateral OFC area 12 in macaques, which then projects to premotor regions (Gerbella *et al.,* 2010; Saleem *et al.,* 2014). Similarly, areas 45 and 44 are thought in humans to project on to premotor cortical regions such as the supplementary motor area (Amunts and Zilles, 2012). Furthermore, area GrFO (an opercular part of area 45 in macaques) could represent a gateway for the access of limbic inputs from for example the lateral orbitofrontal cortex, about subjective values, emotional significance of stimuli or internal states, to the PMv area (ventral premotor area F5a) involved in selecting appropriate goal-directed hand and mouth/face actions (Gerbella *et al.,* 2016). It is of interest that this occurs in the right hemisphere. In the left hemisphere, the corresponding inferior frontal gyrus areas include Broca's area, which in the current context may be thought of as connecting language processing areas in the left hemisphere to premotor cortical areas for output. The functional connectivity that provides for this is different for the inferior frontal gyrus between the right and left hemispheres (Du *et al.,* 2020).

Another finding is confirmation that the lateral orbitofrontal cortex [including in this investigation AAL2 (Rolls *et al.,* 2015) areas lateral orbitofrontal cortex and the inferior frontal gyrus pars orbitalis (IFGOrb), corresponding to area BA12/47 (Ongür and Price, 2000; Öngür *et al.,* 2003)] has higher functional connectivity in depression with areas that include the posterior cingulate cortex (Cheng *et al.,* 2018b) and precuneus (Cheng *et al.,* 2018c). This is especially evident on the right, where the lateral orbitofrontal cortex, unrestricted by Broca's area on the left, extends round the right inferior prefrontal convexity and is contiguous with the right inferior frontal gyrus (Öngür *et al.,* 2003). Now this lateral orbitofrontal cortex area has been implicated in depression because it responds to not receiving expected rewards, which is a typical trigger of sadness and depression (Rolls, 2016b, 2018a, 2019b,c; Xie *et al.,* 2020). The present findings are consistent with that hypothesis, but raise the interesting issue of the relative importance of over-responsiveness to non-reward (which may be related to increased functional connectivity of the lateral orbitofrontal cortex in depression), *vs* over-responsiveness to stop signals for behavior (or too little initiation of action), which may be related to increased functional connectivity of the right inferior frontal gyrus, as described here in depression. In fact, it is likely that under normal circumstances, the detection in the lateral orbitofrontal cortex of non-reward may use as an output to influence behavior the right inferior frontal gyrus (which in turn has connections to premotor cortical areas), so that increased functional connectivity of both the lateral orbitofrontal cortex and the inferior frontal gyrus in depression, as described here, could lead to the reduced motivation to perform actions that is a characteristic of depression.

The higher functional connectivity of the non-reward associated lateral orbitofrontal cortex with the posterior cingulate cortex confirmed here may be related to the increased rumination of sad memories in depression (Cheng *et al.,* 2018b), for the posterior cingulate cortex provides a route into the hippocampal memory system (Rolls and Wirth, 2018; Rolls, 2018b, 2019a); is implicated in episodic memory including autobiographical memory, and imagining the future (Auger and Maguire, 2013; Leech and Sharp, 2014); and has functional connectivity that is related to the rumination in depression (Berman *et al.,* 2011). The higher functional connectivity of the non-reward associated lateral orbitofrontal cortex with the precuneus confirmed here may be related to the poor self-esteem in depression (Cheng *et al.,* 2018c), for the precuneus is implicated in the representations of the self and in autobiographical recall (Cavanna and Trimble, 2006; Freton *et al.,* 2014), and has connections with the posterior cingulate cortex (Vogt, 2009; Rolls, 2019a). This higher functional connectivity of the lateral orbitofrontal cortex related to depression has been confirmed in an independent data set from the USA in which depressive symptoms were associated with higher lateral orbitofrontal cortex functional connectivity (Cheng *et al.,* 2018d).

The lower functional connectivity of the medial orbitofrontal cortex described here in unmedicated patients very usefully extends what was described in a combined group of medicated and unmedicated patients previously (Cheng *et al.,* 2016). We relate (Cheng *et al.,* 2016) the lower functional connectivity of medial orbitofrontal cortex areas, implicated in reward (Rolls, 2016b, 2018a, 2019b,c), with the medial temporal lobe areas, implicated in memory (Rolls, 2016a, 2018b; Rolls and Wirth, 2018), to the reduction in happy memories that is found in depression. However, a surprising result was that medication did not reverse these lower functional connectivities, but instead, these medial orbitofrontal cortex functional connectivities were even lower with medication in depressed patients than they were in controls. This has very interesting implications for new treatments that might treat these reduced medial orbitofrontal cortex functional connectivities in depression, given that current medications appear to reduce the higher functional connectivities in depression but not to normalize the lower functional connectivities.

We have reported previously that the lower functional connectivities of the medial orbitofrontal cortex with medial temporal cortex areas in depression are correlated with the BDI and Hamilton Depression scores (Cheng *et al.,* 2016), so the functional connectivities being investigated are related to depression severity. In addition, we note that in the present investigation, the medication was associated with differences in functional connectivity (Supplementary Figures S4–S7) and with depression scores (Supplementary Table S1), providing further evidence that the functional connectivities described here which are different in depression do relate to the symptoms of depression.

Overall, the results described in this paper provide new evidence on increased functional connectivity in depression of the right inferior frontal gyrus and are consistent with the hypothesis that the right inferior frontal gyrus provides a route for the orbitofrontal cortex [implicated in mood, emotion and depression (Cheng *et al.,* 2016; Rolls, 2016b, 2018a, 2019b)] to connect to output pathways such as premotor cortical areas. An implication is that reducing the activity of the right inferior frontal gyrus as well as of the lateral orbitofrontal cortex may be a useful aim for treatments for depression. In this connection, it is of interest that transcranial magnetic stimulation of lateral prefrontal cortical areas has been reported to be useful in treatment-resistant depression (Feffer *et al.,* 2018). It is also of interest that the right inferior frontal cortex has increased functional connectivity in depression with anterior cingulate cortex areas that have been implicated in depression (Hamani *et al.,* 2011; McGrath *et al.,* 2013; Riva-Posse *et al.,* 2018; Rolls *et al.,* 2019a).

The results obtained here provide new evidence on the brain processes related to depression, in that they provide a voxel-level analysis of functional connectivity of the inferior frontal gyrus for the first time and orbitofrontal cortex in depression, and this level of resolution and a sufficient sample size are important in separating out fundamental differences in the connectivity of the nearby medial orbitofrontal cortex and lateral orbitofrontal cortex, which appear to play complementary roles in depression (Rolls, 2016b, 2019b, c). The voxel-level analysis implemented here goes far beyond previous brain region or network-level investigations, for it shows which voxels in the orbitofrontal cortex and inferior frontal gyrus have different functional connectivity in depression, and also which voxels in the connected different brain areas have the different functional connectivity in depression. This voxel-level analysis allows us for example to separate out different connectivity changes in the nearby medial and lateral orbitofrontal cortex, and right and left inferior frontal gyrus. This voxel-level analysis is only possible because of the large number of individuals included in this investigation, for that enables voxel-level results to be statistically significant even when corrected for the large number of multiple comparisons. Moreover, the results here are new; in that, they focus on connectivity in these areas in unmedicated patients and then show the differences that are related to medication. The complementarity is that as shown here in unmedicated patients, the medial orbitofrontal cortex, implicated in reward, has reduced functional connectivity in depression; the lateral orbitofrontal cortex, implicated in non-reward and therefore in negative emotions, has increased functional connectivity in depression (Rolls, 2016b, 2019b,c). The research described here also implicates the right inferior frontal gyrus in depression, as an area that provides a route for the lateral orbitofrontal cortex to influence behavior by the onward connections of the inferior frontal gyrus to premotor cortical areas. Consistently, this inferior frontal gyrus region appears to have altered activations to face expressions that are related to major depressive disorder (Briceno *et al.,* 2013; Jenkins *et al.,* 2016). Moreover, a role for the right inferior frontal gyrus in depression may well be related to the fact that if lesions of this region lead to a failure to inhibit behavior, and to increased impulsiveness (Aron *et al.,* 2014), then over-efficacy of the inferior frontal gyrus (for example involving the high functional connectivity described here) may contribute to too little initiation of behavior and action, which is a hallmark of depression.

## Contributors

Edmund T. Rolls, Wei Cheng and Jianfeng Feng contributed to the design of the study. Dongtao Wei, Jiang Qiu and Peng Xie contributed to the collection of the data. Wei Cheng, Edmund T. Rolls and Jingnan Du contributed to the analysis of the data and the preparation of the manuscript. Edmund T. Rolls and Wei Cheng participated in writing the paper. All collaborators had an opportunity to discuss the research and contribute to the paper.

## Funding

J. F. was supported by the 111 Project (No. B18015), the key project of Shanghai Science & Technology (No. 16JC1420402), the National Key R&D Program of China (No. 2018YFC1312900), the National Natural Science Foundation of China (NSFC 91630314), the Shanghai Municipal Science and Technology Major Project (No. 2018SHZDZX01) and ZJLab. W.C. was supported by grants from the National Natural Sciences Foundation of China (No. 81701773, 11771010), Sponsored by Shanghai Sailing Program (No. 17YF1426200) and the Research Fund for the Doctoral Program of Higher Education of China (No. 2017M610226). W.C. was also sponsored by the Natural Science Foundation of Shanghai (No. 18ZR1404400). J.Q. was supported by the National Natural Science Foundation of China (31271087; 31470981; 31571137; 31500885), the National Outstanding young people plan, the Program for the Top Young Talents by Chongqing, the Fundamental Research Funds for the Central Universities (SWU1509383), the Natural Science Foundation of Chongqing (cstc2015jcyjA10106) and General Financial Grant from the China Postdoctoral Science Foundation (2015M572423). P.X. was supported by the National Key R&D Program of China (2017YFA0505700). D.Y. was supported by the National Natural Science Foundation of China (grant no. 31300917), Chongqing Research Program of Basic Research and Frontier Technology (No. cstc2016jcyjA0352).

## Conflict of interest

All authors declare no competing financial interests.

## Supplementary Material

scan-19-310-File002_nsaa014
