# Supplementary material for: Functional connectivity of the right inferior frontal gyrus and orbitofrontal cortex in depression
Source: Soc Cogn Affect Neurosci. 2020 Jan 28;15(1):75–86. doi: 10.1093/scan/nsaa014 (PMC7171374; doi:10.1093/scan/nsaa014)
Supplement: scan-19-310-File002_nsaa014 [file scan-19-310-file002_nsaa014.docx]

**Functional connectivity of the right inferior frontal gyrus and orbitofrontal cortex in depression**

**Supplementary Material**

Edmund T. Rolls^1,2,3,#^; Wei Cheng^1,#,*^; Jingnan Du^1,#^; Dongtao Wei^5,#^; Jiang Qiu^4,5,#^; Dan Dai^1,#^; Qunjie Zhou^1,#^; Peng Xie^8,9,10,*^; Jianfeng Feng^1, 2, 12,*^

1. Institute of Science and Technology for Brain-inspired Intelligence, Fudan University, Shanghai, 200433, China

2. Department of Computer Science, University of Warwick, Coventry CV4 7AL, UK

3. Oxford Centre for Computational Neuroscience, Oxford, UK

4. Key Laboratory of Cognition and Personality (SWU), Ministry of Education, Chongqing, China

5. Department of Psychology, Southwest University, Chongqing, China

6. Institute of Neuroscience, Chongqing Medical University, Chongqing, China

7. Chongqing Key Laboratory of Neurobiology, Chongqing, China

8. Department of Neurology, Yongchuan Hospital of Chongqing Medical University, Chongqing 402160, China

9. School of Mathematical Sciences, School of Life Science and the Collaborative Innovation Center for Brain Science, Fudan University, Shanghai, 200433, PR China

^#^ These authors contributed equally to this work.

* Corresponding authors

**Participants**

There were 282 patients with a diagnosis of major depression, and 254 controls. The patients were from Xinan (First Affiliated Hospital of Chongqing Medical School in Chongqing, China). All participants were diagnosed according to the Diagnostic and Statistical Manual of Mental Disorder-IV criteria for major depressive disorder. Depression severity and symptomatology were evaluated by the Hamilton Depression Rating Scale (HAMD, 17 items) (Hamilton, 1960) and the Beck Depression Inventory (BDI) (Beck and Beamesderfer, 1974). Table S1 provides a summary of the demographic information and the psychiatric diagnosis (showing how they were diagnosed) of the participants. The data collection was approved by the local ethical review committees, was in accordance with the Code of Ethics of the World Medical Association (Declaration of Helsinki), and informed consent was obtained. This is a subset of patients from a previous functional connectivity investigation that can now be analyzed further (Cheng *et al.*, 2016), and the analysis used here is completely different and novel in its application to depression, in the ways set out in the paper. With respect to age and sex, Table S1 shows that there were no significant differences in the age and sex of the depressed groups and the controls. Further, the effects of age and sex were regressed out in all analyses. 125 of the patients were not receiving medication at the time of the neuroimaging. The patients receiving medication were different participants. The medication for these patients consisted in most cases of selective serotonin reuptake inhibitors (SSRIs) including fluoxetine, paroxetine, sertraline, citalopram and escitalopram; or serotonin-norepinephrine reuptake inhibitors (SNRIs) such as venflaxine, or a tetracyclic antidepressant such as mirtazepine. Further details follow.

Patients with MDD were recruited from the outpatient department of the First Affiliated Hospital of Chongqing Medical School in Chongqing, China. All were diagnosed according to the Structured Clinical Interview for DSM-IV, by independent assessments of two psychiatrists. They were also assessed for disease severity using the Hamilton Depression Rating Scale (HAMD) (Hamilton, 1960) and Beck Depression Inventory (BDI), illness duration and the medication status of the patients. Before the investigation, we excluded individuals who were not suitable for MRI scanning by interview and by the self-reported checklist. The MRI related exclusion criteria include claustrophobia, metallic implants, Meniere’s Syndrome and a history of fainting within the previous half year. Exclusion criteria for both groups were as follows: current psychiatric disorders (except for MDD) and neurological disorders; substance abuse; and stroke or serious encephalopathy. Of note, all of the subjects in the control group did not meet DSM-IV criteria for any psychiatric disorders and did not use any drugs that could affect brain function. This study was approved by the Research Ethics Committee of the Brain Imaging Center of Southwest University and First Affiliated Hospital of Chongqing Medical School. Informed written consent was obtained from each subject. This study was conducted in accordance with the Helsinki Declaration as revised in 1989.

**Image Acquisition**

All images were acquired on a 3.0-T Siemens Trio MRI scanner using a 16-channel whole-brain coil (Siemens Medical, Erlangen, Germany). High- resolution T1-weighted 3D images were acquired using a magnetization-prepared rapid gradient echo (MPRAGE) sequence (echo time (TE) = 2.52 ms; repetition time (TR) = 1900 ms; inversion time (TI) = 900 ms; flip angle = 9 degrees; slices = 176; thickness = 1.0 mm; resolution matrix = 256×256; voxel size = 1×1×1 mm3). For each participant, 242 functional images were acquired with a gradient echo type Echo Planar Imaging (EPI) sequence (echo time (TE) = 30 ms; repetition time (TR) = 2000 ms; flip angle = 90 degrees; slices = 32; slice thickness = 3.0 mm; slice gap = 1 mm; resolution matrix = 64×64; voxel size 3.4×3.4×3mm^3^). Data for resting state functional connectivity analysis were collected in an 8 min period in which the participants were awake in the scanner not performing a task using a standard protocol in which the participants were asked to look at a white fixation point on a dark background. All participants performed this during the fMRI imaging as confirmed by the participants after the session.

**Table S1.** A summary of the demographic information and the psychiatric diagnosis in the present study.

| **Group** | **Age (years)** | **Sex (male/female)** | **Education (years)** | **Medication (yes / no)** | **HAMD** | **BDI** | **Duration of illness** | **First episode (yes / no)** | **Mean FD** |
| --- | --- | --- | --- | --- | --- | --- | --- | --- | --- |
| Healthy | 39.65 ± 15.80 | 166 / 88 | 13.01 ± 3.89 | / | / | / | / | / | 0.133 ±0.063 |
| Patient | 38.74 ± 13.65 | 183 / 99 | 11.91 ± 3.58 | 157 / 125 | 20.8 ± 5.87 | 20.42 ± 9.33 | 4.16 ± 5.51 | 209 / 49 | 0.125 ± 0.054 |
| Statistic  (t / p) or (chi-square / p) | 0.719 / 0.472 | 0.013 / 0.911 | 3.41 / 6.9e-4 | / | / | / | / | / | 1.729 / 0.084 |
| Unmedicated  patient | 37.60 ± 13.12 | 84 / 41 | 12.07 ± 3.72 | 125 / 0 | 22.22 ± 4.39 | 22.51 ± 8.16 | 2.91 ± 4.44 | 111 / 14 | 0.120 ± 0.053 |
| Medicated  patient | 39.64 ± 14.03 | 99 / 58 | 11.78 ± 3.48 | 0 / 157 | 19.42 ± 6.73 | 18.43 ± 9.95 | 5.33 ± 6.13 | 98 / 35 | 0.129 ± 0.054 |
| Statistic  (t / p) or (chi-square / p) | -1.250 / 0.212 | 0.524 / 0.469 | 0.673 / 0.501 | / | 3.907 / 1.2e-4 | 3.520 / 5.1e-4 | -3.539 / 4.8e-4 | 9.570 / 0.002 | -1.268 / 0.206 |

Values are n or mean ± SD.

Note: The difference between patients and controls for continuous variables was assessed by a two-sample t-test and the difference for the binary variable (gender) was assessed by a chi-square test.

**Table S2a.** The differences in functional connectivity in unmedicated patients with depression of the voxels in the brain areas shown in the columns with other brain areas (shown in the rows). This is the number of voxels found with p<0.05 FDR. The names are from the AAL2 atlas (Rolls *et al.*, 2015).

| **Lateral orbitofrontal cortex** | | | | | **Medial orbitofrontal cortex** | | | | | |
| --- | --- | --- | --- | --- | --- | --- | --- | --- | --- | --- |
| **Region** | **Change pattern** | **# voxels** | **Peak value** | **MNI cordinates** | **Region** | **Change pattern** | **# voxels** | **Peak value** | **MNI cordinates** |  |
| Precuneus | Higher | 357 | 190.83 | [3,-54,36] | Rectus | Higher | 375 | 1027.60 | [-6,39,-27] |  |
|  | Lower | 0 | 0.00 | [90,-126,-72] |  | Lower | 139 | -567.75 | [15,30,-21] |  |
| Frontal_Inf_Orb_2 | Higher | 101 | 2083.53 | [33,33,-12] | Precuneus | Higher | 343 | 104.25 | [3,-54,36] |  |
|  | Lower | 8 | -340.19 | [-21,27,-12] |  | Lower | 0 | 0.00 | [90,-126,-72] |  |
| Cingulate_Post | Higher | 94 | 95.25 | [6,-54,30] | Orbitofrontal Cortex Med | Higher | 124 | 712.78 | [-9,36,-27] |  |
|  | Lower | 0 | 0.00 | [90,-126,-72] |  | Lower | 167 | -536.07 | [18,30,-21] |  |
| Supp_Motor_Area | Higher | 0 | 0.00 | [90,-126,-72] | Orbitofrontal Cortex Post | Higher | 142 | 2075.34 | [36,33,-15] |  |
|  | Lower | 83 | -13.49 | [0,15,54] |  | Lower | 88 | -412.80 | [-21,27,-15] |  |
| Frontal_Med_Orb | Higher | 64 | 34.76 | [0,51,-15] | Frontal_Mid_2 | Higher | 181 | 564.71 | [42,21,33] |  |
|  | Lower | 0 | 0.00 | [90,-126,-72] |  | Lower | 12 | -8.46 | [-39,36,42] |  |
| Frontal_Mid_2 | Higher | 52 | 126.82 | [36,24,21] | Temporal_Inf | Higher | 0 | 0.00 | [90,-126,-72] |  |
|  | Lower | 0 | 0.00 | [90,-126,-72] |  | Lower | 185 | -274.81 | [-39,-12,-39] |  |
| Orbitofrontal Cortex Lat | Higher | 49 | 927.69 | [42,36,-18] | Frontal_Sup_2 | Higher | 162 | 262.54 | [15,45,42] |  |
|  | Lower | 3 | -8.31 | [-48,33,-15] |  | Lower | 11 | -25.30 | [-15,0,69] |  |
| Calcarine | Higher | 37 | 40.67 | [3,-60,18] | Orbitofrontal Cortex Ant | Higher | 63 | 1644.28 | [36,36,-15] |  |
|  | Lower | 0 | 0.00 | [90,-126,-72] |  | Lower | 105 | -209.93 | [-18,33,-15] |  |
| Angular | Higher | 32 | 52.94 | [-39,-57,27] | Frontal_Sup_Medial | Higher | 132 | 205.92 | [15,45,45] |  |
|  | Lower | 0 | 0.00 | [90,-126,-72] |  | Lower | 3 | -4.37 | [12,66,27] |  |
| Frontal_Sup_Medial | Higher | 21 | 43.23 | [-3,39,42] | Caudate | Higher | 109 | 149.84 | [21,18,6] |  |
|  | Lower | 8 | -8.37 | [-3,30,45] |  | Lower | 18 | -76.40 | [15,24,-3] |  |
| Cingulate_Mid | Higher | 27 | 123.72 | [3,-51,33] | Temporal_Pole_Mid | Higher | 0 | 0.00 | [90,-126,-72] |  |
|  | Lower | 0 | 0.00 | [90,-126,-72] |  | Lower | 113 | -413.75 | [24,6,-36] |  |
| Cingulate_Ant | Higher | 19 | 42.81 | [-12,45,-3] | ParaHippocampal | Higher | 0 | 0.00 | [90,-126,-72] |  |
|  | Lower | 0 | 0.00 | [90,-126,-72] |  | Lower | 97 | -609.82 | [24,3,-30] |  |
| Precentral | Higher | 17 | 39.31 | [45,3,39] | Cingulate_Post | Higher | 90 | 91.09 | [-6,-51,27] |  |
|  | Lower | 0 | 0.00 | [90,-126,-72] |  | Lower | 0 | 0.00 | [90,-126,-72] |  |
| Temporal_Inf | Higher | 0 | 0.00 | [90,-126,-72] | Supp_Motor_Area | Higher | 0 | 0.00 | [90,-126,-72] |  |
|  | Lower | 12 | -18.09 | [-42,0,-36] |  | Lower | 73 | -117.26 | [-12,0,63] |  |
| Cuneus | Higher | 11 | 30.67 | [0,-66,24] | Fusiform | Higher | 0 | 0.00 | [90,-126,-72] |  |
|  | Lower | 0 | 0.00 | [90,-126,-72] |  | Lower | 72 | -243.97 | [-36,-12,-42] |  |
| Frontal_Sup_2 | Higher | 2 | 4.21 | [-24,39,27] | Frontal_Med_Orb | Higher | 68 | 82.54 | [6,51,-6] |  |
|  | Lower | 4 | -4.42 | [-15,0,69] |  | Lower | 2 | -4.17 | [6,24,-15] |  |
| Occipital_Mid | Higher | 3 | 21.72 | [-33,-63,30] | Calcarine | Higher | 59 | 84.05 | [3,-60,18] |  |
|  | Lower | 0 | 0.00 | [90,-126,-72] |  | Lower | 0 | 0.00 | [90,-126,-72] |  |
| Hippocampus | Higher | 2 | 8.30 | [-24,-21,-15] | Cingulate_Ant | Higher | 51 | 115.58 | [12,42,24] |  |
|  | Lower | 0 | 0.00 | [90,-126,-72] |  | Lower | 0 | 0.00 | [90,-126,-72] |  |
| Lingual | Higher | 2 | 16.71 | [9,-42,6] | Postcentral | Higher | 1 | 8.61 | [30,-42,75] |  |
|  | Lower | 0 | 0.00 | [90,-126,-72] |  | Lower | 48 | -34.26 | [-63,-9,21] |  |
| Parietal_Sup | Higher | 2 | 4.21 | [-24,-72,60] | Cingulate_Mid | Higher | 40 | 107.76 | [3,-51,33] |  |
|  | Lower | 0 | 0.00 | [90,-126,-72] |  | Lower | 8 | -33.74 | [-9,-9,48] |  |

**Table S2b.** The differences in functional connectivity in in unmedicated patients with depression of the voxels in the brain areas shown (Inferior Frontal Gyrus pars triangularis and pars opercularis) in the columns with other brain areas (shown in the rows). This is the number of voxels found with p<0.05 FDR.

| **IFGtri** | | | | | **IFGoperc** | | | | |
| --- | --- | --- | --- | --- | --- | --- | --- | --- | --- |
| **Region** | **Change pattern** | **# voxels** | **Peak value** | **MNI cordinates** | **Region** | **Change pattern** | **# voxels** | **Peak value** | **MNI coordinates** |
| Precentral | Higher | 1175 | 229.72 | [-54,-63,18] | Temporal_Mid | Higher | 1159 | 409.01 | [54,-3,-24] |
|  | Lower | 0 | 0 | [90,-126,-72] |  | Lower | 0 | 0.00 | [90,-126,-72] |
| Frontal_Sup_2 | Higher | 961 | 197.54 | [-6,45,18] | Precuneus | Higher | 799 | 375.89 | [-3,-54,33] |
|  | Lower | 2 | -4.16 | [12,33,60] |  | Lower | 0 | 0.00 | [90,-126,-72] |
| Frontal_Mid_2 | Higher | 872 | 170.69 | [-15,54,9] | Frontal_Sup_Medial | Higher | 527 | 197.01 | [15,45,6] |
|  | Lower | 27 | -38.37 | [-18,-3,54] |  | Lower | 0 | 0.00 | [90,-126,-72] |
| Frontal_Inf_Oper | Higher | 617 | 164.38 | [-3,-57,33] | Frontal_Inf_Oper | Higher | 423 | 13997.5 | [39,15,30] |
|  | Lower | 1 | -4.09 | [9,-60,51] |  | Lower | 5 | -8.24 | [-33,12,30] |
| Frontal_Inf_Tri | Higher | 460 | 21272.47 | [36,15,27] | Frontal_Sup_2 | Higher | 322 | 137.72 | [-15,54,9] |
|  | Lower | 34 | -114.83 | [-42,45,12] |  | Lower | 0 | 0.00 | [90,-126,-72] |
| Frontal_Inf_Orb_2 | Higher | 390 | 164.28 | [-57,-9,-36] | Angular | Higher | 248 | 366.28 | [-45,-69,27] |
|  | Lower | 7 | -16.94 | [-45,-21,-30] |  | Lower | 0 | 0.00 | [90,-126,-72] |
| Rolandic_Oper | Higher | 387 | 290.16 | [-45,-69,27] | Temporal_Inf | Higher | 215 | 276.03 | [-60,-9,-27] |
|  | Lower | 0 | 0 | [90,-126,-72] |  | Lower | 0 | 0.00 | [90,-126,-72] |
| Supp_Motor_Area | Higher | 342 | 146.58 | [0,57,-3] | Temporal_Pole_Sup | Higher | 214 | 421.37 | [-48,9,-24] |
|  | Lower | 0 | 0 | [90,-126,-72] |  | Lower | 0 | 0.00 | [90,-126,-72] |
| Olfactory | Higher | 334 | 168.66 | [-12,45,0] | Frontal_Med_Orb | Higher | 207 | 323.99 | [0,51,-15] |
|  | Lower | 0 | 0 | [90,-126,-72] |  | Lower | 0 | 0.00 | [90,-126,-72] |
| Frontal_Sup_Medial | Higher | 226 | 312.86 | [42,-39,57] | Cingulate_Post | Higher | 185 | 382.13 | [0,-54,30] |
|  | Lower | 0 | 0 | [90,-126,-72] |  | Lower | 0 | 0.00 | [90,-126,-72] |
| Frontal_Med_Orb | Higher | 221 | 137.27 | [-6,-48,33] | Cingulate_Ant | Higher | 181 | 153.79 | [15,42,6] |
|  | Lower | 0 | 0 | [90,-126,-72] |  | Lower | 0 | 0.00 | [90,-126,-72] |
| Rectus | Higher | 208 | 177.94 | [-48,9,-21] | Cingulate_Mid | Higher | 178 | 286.61 | [-6,-48,33] |
|  | Lower | 0 | 0 | [90,-126,-72] |  | Lower | 0 | 0.00 | [90,-126,-72] |
| Oorbitofrontal Cortex Med | Higher | 203 | 144.73 | [-42,15,-30] | Temporal_Pole_Mid | Higher | 130 | 392.01 | [-42,15,-30] |
|  | Lower | 0 | 0 | [90,-126,-72] |  | Lower | 1 | -4.15 | [-18,6,-36] |
| Orbitofrontal Cortex Ant | Higher | 182 | 168.17 | [-3,-51,30] | Calcarine | Higher | 84 | 214.59 | [3,-60,18] |
|  | Lower | 0 | 0 | [90,-126,-72] |  | Lower | 0 | 0.00 | [90,-126,-72] |
| Orbitofrontal Cortex Post | Higher | 156 | 115.89 | [-57,-57,24] | Hippocampus | Higher | 83 | 288.75 | [33,-6,-21] |
|  | Lower | 0 | 0 | [90,-126,-72] |  | Lower | 0 | 0.00 | [90,-126,-72] |
| Orbitofrontal Cortex Lat | Higher | 142 | 124.09 | [15,0,69] | Postcentral | Higher | 76 | 103.17 | [42,-39,57] |
|  | Lower | 2 | -25.66 | [15,6,51] |  | Lower | 0 | 0.00 | [90,-126,-72] |
| Insula | Higher | 141 | 95.42 | [54,-57,21] | Temporal_Sup | Higher | 69 | 179.78 | [-57,0,-15] |
|  | Lower | 0 | 0 | [90,-126,-72] |  | Lower | 0 | 0.00 | [90,-126,-72] |
| Cingulate_Ant | Higher | 139 | 108.59 | [42,30,21] | Frontal_Mid_2 | Higher | 59 | 187.03 | [-33,27,21] |
|  | Lower | 0 | 0 | [90,-126,-72] |  | Lower | 0 | 0.00 | [90,-126,-72] |
| Cingulate_Mid | Higher | 76 | 60.41 | [33,-9,-21] | Cuneus | Higher | 58 | 192.40 | [-3,-66,21] |
|  | Lower | 0 | 0 | [90,-126,-72] |  | Lower | 0 | 0.00 | [90,-126,-72] |
| Cingulate_Post | Higher | 60 | 87.51 | [-3,-66,24] | Supp_Motor_Area | Higher | 46 | 47.31 | [-9,-9,75] |
|  | Lower | 0 | 0 | [90,-126,-72] |  | Lower | 0 | 0.00 | [90,-126,-72] |

**Table S3.** The anatomical regions defined in each hemisphere and their label in the automated anatomical labelling atlas AAL2 (Rolls *et al.*, 2015). Column 4 provides a set of possible abbreviations for the anatomical descriptions.

| NO. | ANATOMICAL DESCRIPTION | LABEL  aal2.nii.gz | POSSIBLE  ABBREVIATION |
| --- | --- | --- | --- |
| 1,2 | Precentral gyrus | Precentral | PreCG |
| 3, 4 | Superior frontal gyrus, dorsolateral | Frontal_Sup | SFG |
| 5, 6 | Middle frontal gyrus | Frontal_Mid | MFG |
| 7, 8 | Inferior frontal gyrus, opercular part | Frontal_Inf_Oper | IFGoperc |
| 9, 10 | Inferior frontal gyrus, triangular part | Frontal_Inf_Tri | IFGtriang |
| 11, 12 | IFG pars orbitalis, | Frontal_Inf_Orb | IFGorb |
| 13, 14 | Rolandic operculum | Rolandic_Oper | ROL |
| 15, 16 | Supplementary motor area | Supp_Motor_Area | SMA |
| 17, 18 | Olfactory cortex | Olfactory | OLF |
| 19, 20 | Superior frontal gyrus, medial | Frontal_Sup_Med | SFGmedial |
| 21, 22 | Superior frontal gyrus, medial orbital | Frontal_Med_Orb | PFCventmed |
| 23, 24 | Gyrus rectus | Rectus | REC |
| 25, 26 | Medial orbital gyrus | OFCmed | OFCmed |
| 27, 28 | Anterior orbital gyrus | OFCant | OFCant |
| 29, 30 | Posterior orbital gyrus | OFCpost | OFCpost |
| 31, 32 | Lateral orbital gyrus | OFClat | OFClat |
| 33, 34 | Insula | Insula | INS |
| 35, 36 | Anterior cingulate & paracingulate gyri | Cingulate_Ant | ACC |
| 37, 38 | Middle cingulate & paracingulate gyri | Cingulate_Mid | MCC |
| 39, 40 | Posterior cingulate gyrus | Cingulate_Post | PCC |
| 41, 42 | Hippocampus | Hippocampus | HIP |
| 43, 44 | Parahippocampal gyrus | ParaHippocampal | PHG |
| 45, 46 | Amygdala | Amygdala | AMYG |
| 47, 48 | Calcarine fissure and surrounding cortex | Calcarine | CAL |
| 49, 50 | Cuneus | Cuneus | CUN |
| 51, 52 | Lingual gyrus | Lingual | LING |
| 53, 54 | Superior occipital gyrus | Occipital_Sup | SOG |
| 55, 56 | Middle occipital gyrus | Occipital_Mid | MOG |
| 57, 58 | Inferior occipital gyrus | Occipital_Inf | IOG |
| 59, 60 | Fusiform gyrus | Fusiform | FFG |
| 61, 62 | Postcentral gyrus | Postcentral | PoCG |
| 63, 64 | Superior parietal gyrus | Parietal_Sup | SPG |
| 65, 66 | Inferior parietal gyrus, excluding supramarginal and angular gyri | Parietal_Inf | IPG |
| 67, 68 | SupraMarginal gyrus | SupraMarginal | SMG |
| 69, 70 | Angular gyrus | Angular | ANG |
| 71, 72 | Precuneus | Precuneus | PCUN |
| 73, 74 | Paracentral lobule | Paracentral_Lobule | PCL |
| 75, 76 | Caudate nucleus | Caudate | CAU |
| 77, 78 | Lenticular nucleus, Putamen | Putamen | PUT |
| 79, 80 | Lenticular nucleus, Pallidum | Pallidum | PAL |
| 81, 82 | Thalamus | Thalamus | THA |
| 83, 84 | Heschl’s gyrus | Heschl | HES |
| 85, 86 | Superior temporal gyrus | Temporal_Sup | STG |
| 87, 88 | Temporal pole: superior temporal gyrus | Temporal_Pole_Sup | TPOsup |
| 89, 90 | Middle temporal gyrus | Temporal_Mid | MTG |
| 91, 92 | Temporal pole: middle temporal gyrus | Temporal_Pole_Mid | TPOmid |
| 93, 94 | Inferior temporal gyrus | Temporal_Inf | ITG |

**Figure S1.** The regions of interest within which the functional connectivity of voxels was analyzed in this investigation. The medial orbitofrontal cortex regions (purple) include from the automated anatomical labelling atlas 2 (Rolls *et al.*, 2015) gyrus rectus, OFCmed, OFCant, and OFCpost. The lateral orbitofrontal cortex (red, approximately BA 47/12) includes OFClat and FrontalInfOrb, with these names shown in Table S3 from the AAL2 atlas. The inferior frontal cortex pars triangularis (approximately BA 45) is in blue, and the inferior frontal cortex pars opercularis (approximately BA 44) is in green.


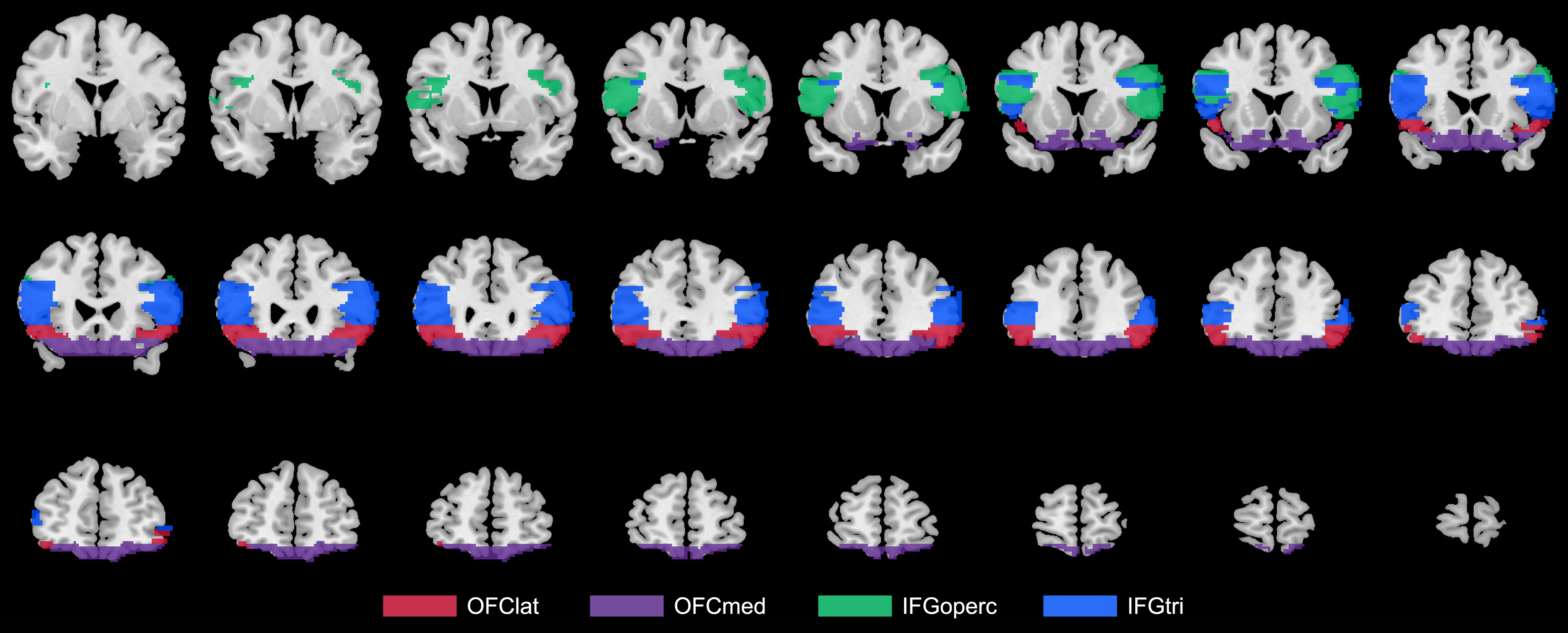


**Figure S2.** Anatomical location of voxels with significantly higher (A) and lower (B) functional connectivity with the inferior frontal gyrus and the orbitofrontal cortex in non-medicated depression (patients - controls) obtained from the voxel-based Association Study. Blue indicates voxels with lower functional connectivity in depressed patients, and red/yellow indicates voxels with higher functional connectivity. In this and in all other Figures, the level of statistical significance for the difference in functional connectivity for any voxel after correction for multiple comparisons was p<0.05 FDR.


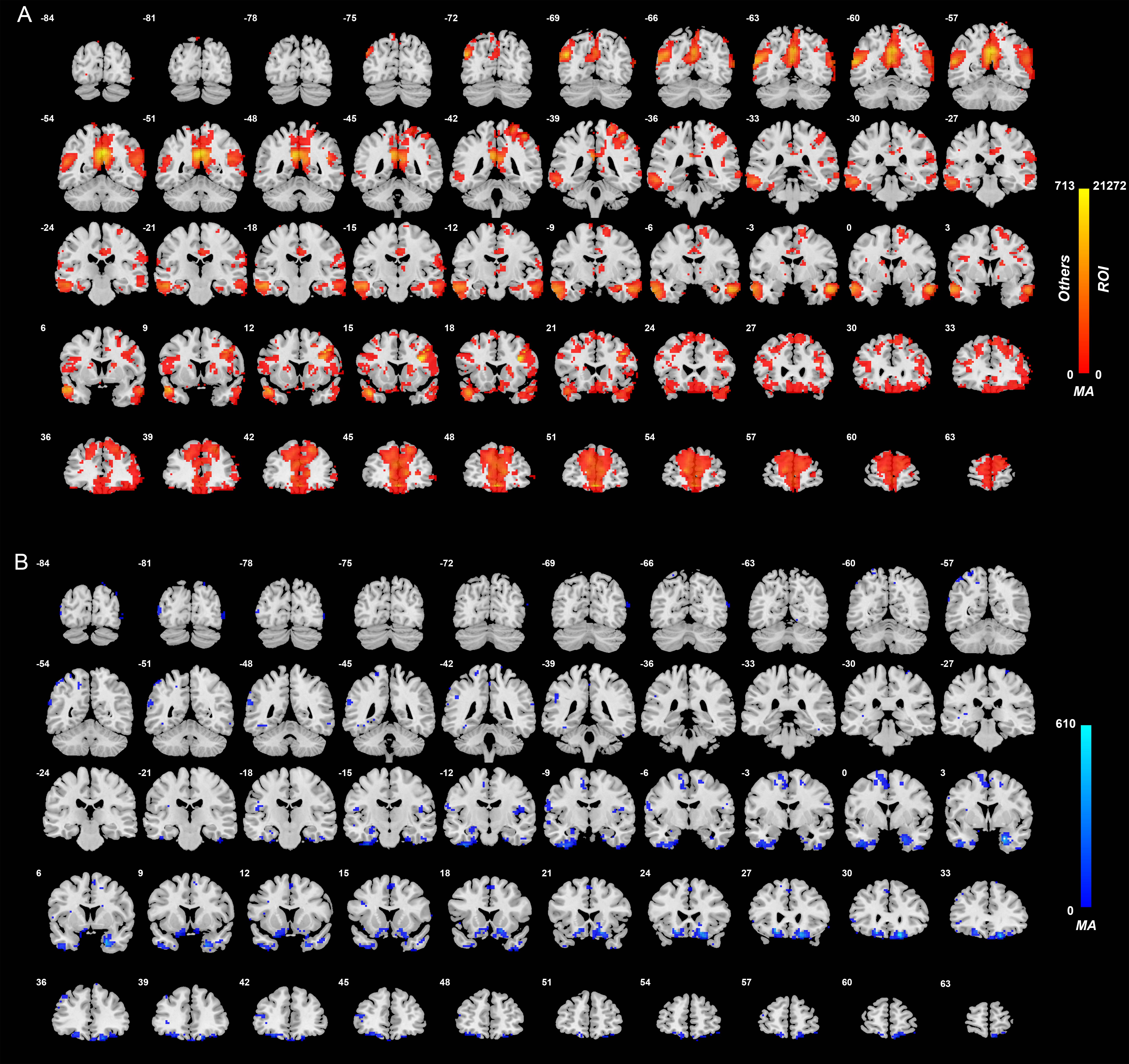


**Figure S3.** Anatomical location of voxels with significantly higher (A) and lower (B) functional connectivity with the inferior frontal gyrus and the orbitofrontal cortex in medicated patients - non-medicated patients obtained from the voxel-based Association Study. Blue indicates voxels with lower functional connectivity in medicated depressed patients, and red/yellow indicates voxels with higher functional connectivity in medicated depressed patients.


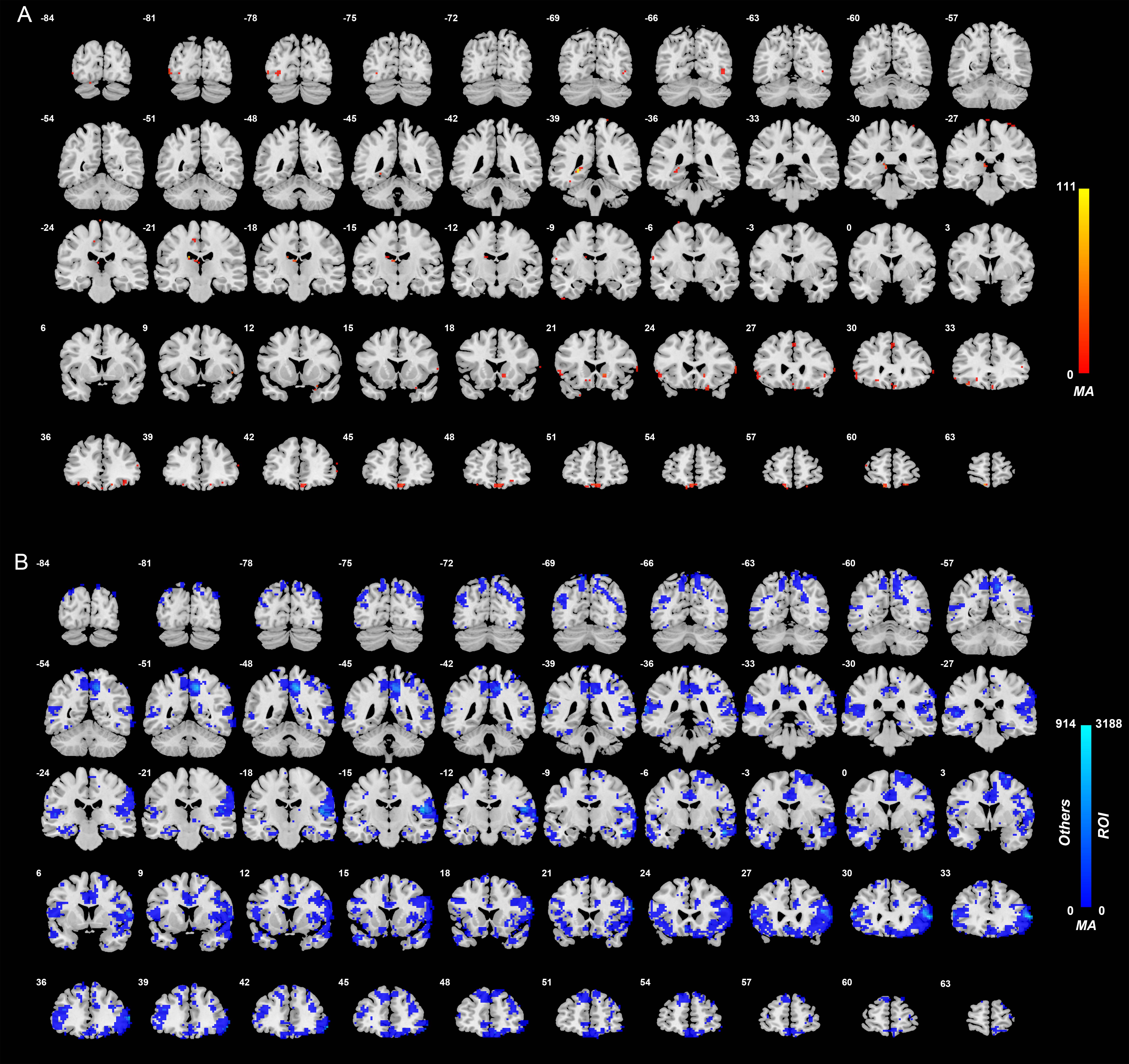


**Figure S4.** Anatomical location of voxels with significantly higher (A) and lower (B) functional connectivity with the lateral orbitofrontal cortex areas in medicated patients - non-medicated patients obtained from the voxel-based Association Study. Blue indicates voxels with lower functional connectivity in medicated than medicated depressed patients, and red/yellow indicates voxels with higher functional connectivity in medicated than medicated depressed patients.


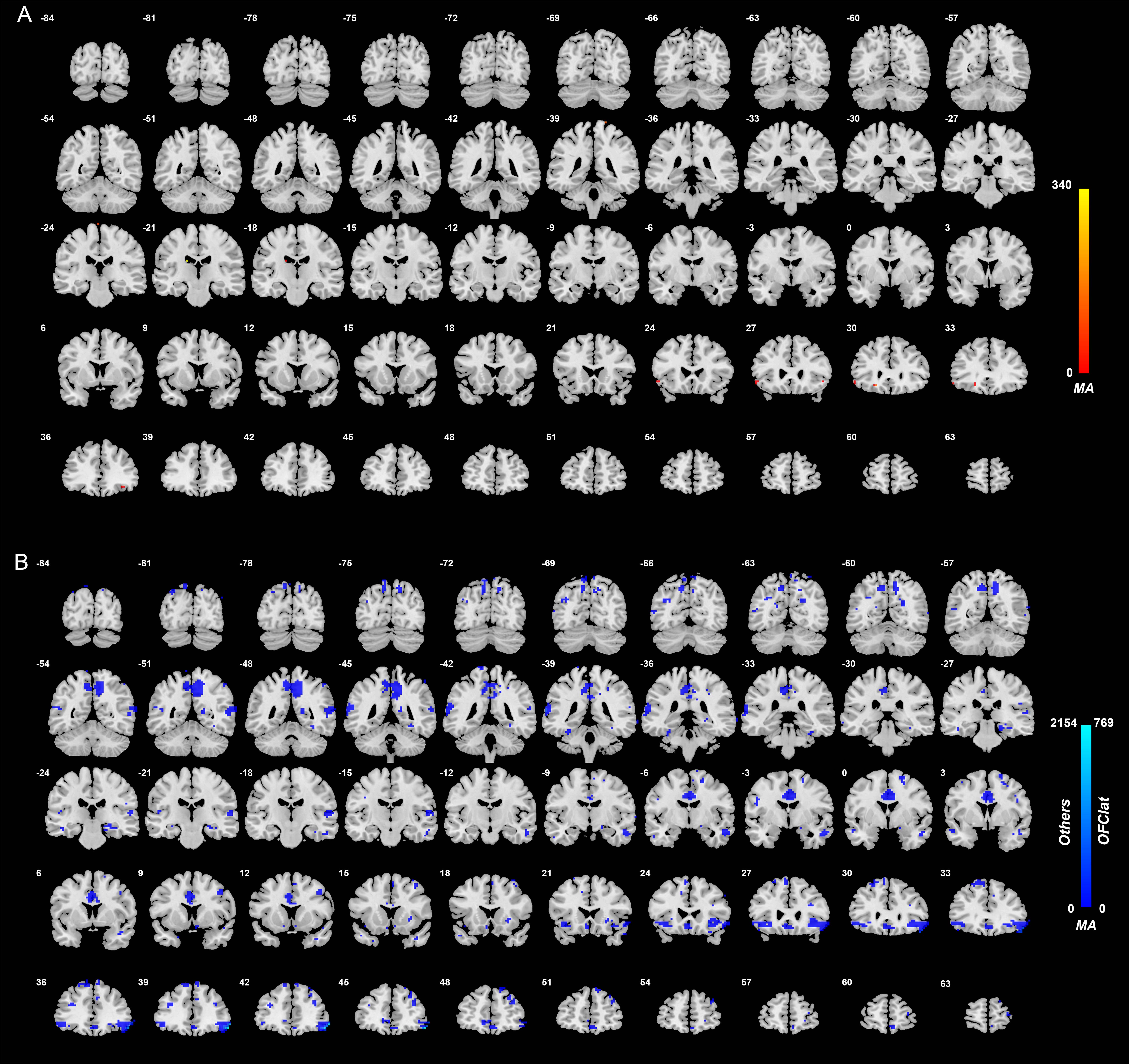


**Figure S5.** Anatomical location of voxels with significantly higher (A) and lower (B) functional connectivity with the medial orbitofrontal cortex areas in medicated patients - non-medicated patients obtained from the voxel-based Association Study. Blue indicates voxels with lower functional connectivity in medicated depressed patients, and red/yellow indicates voxels with higher functional connectivity in medicated depressed patients.


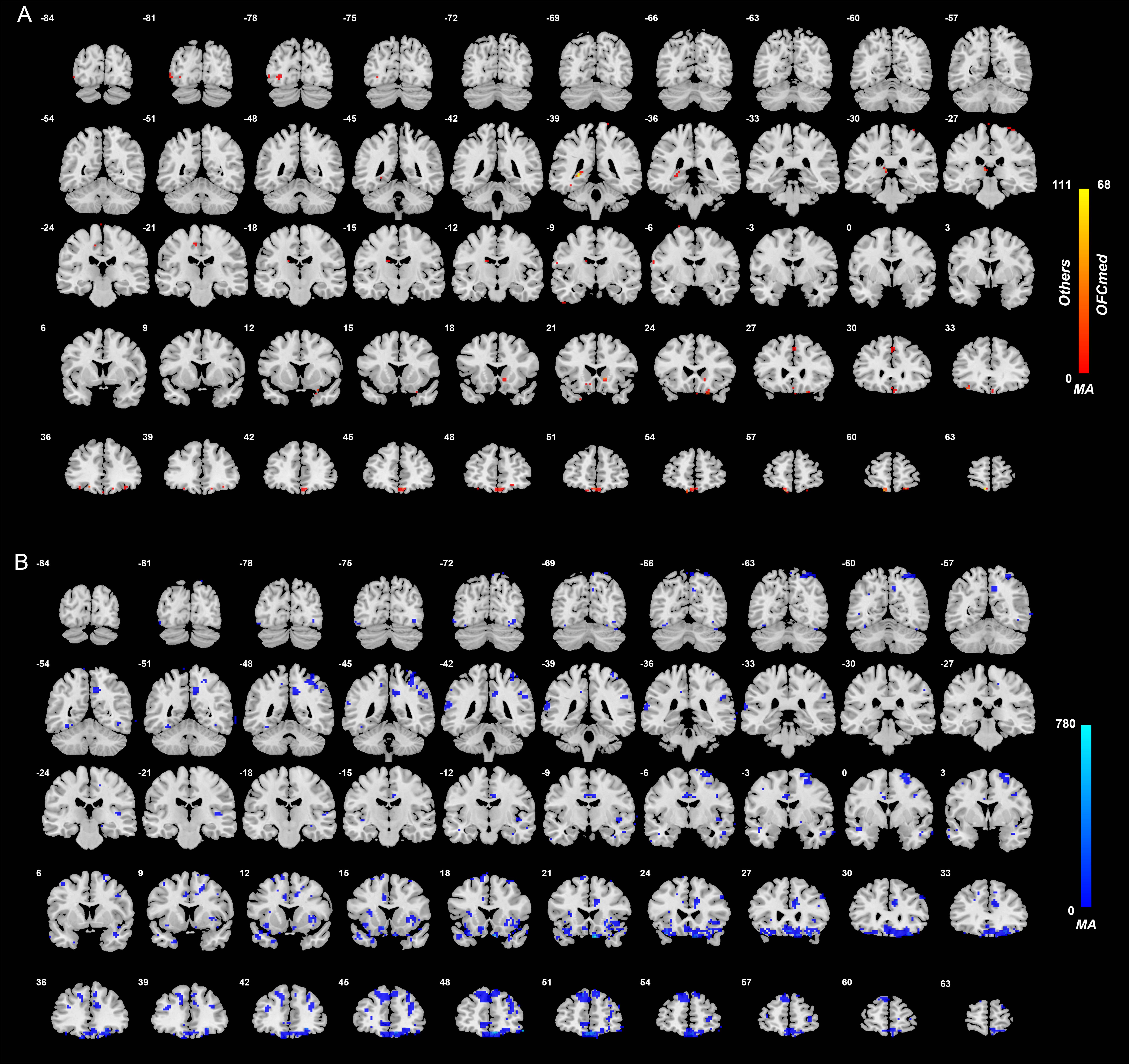


**Figure S6.** Anatomical location of voxels with significantly higher (A) and lower (B) functional connectivity of the inferior frontal gyrus (triangular part) in medicated patients - non-medicated patients obtained from the voxel-based Association Study. Blue indicates voxels with lower functional connectivity in medicated depressed patients, and red/yellow indicates voxels with higher functional connectivity in medicated depressed patients. Medication was associated with lower functional connectivity with the precuneus, motor cortical areas, the temporal cortex, and the supramarginal gyrus.


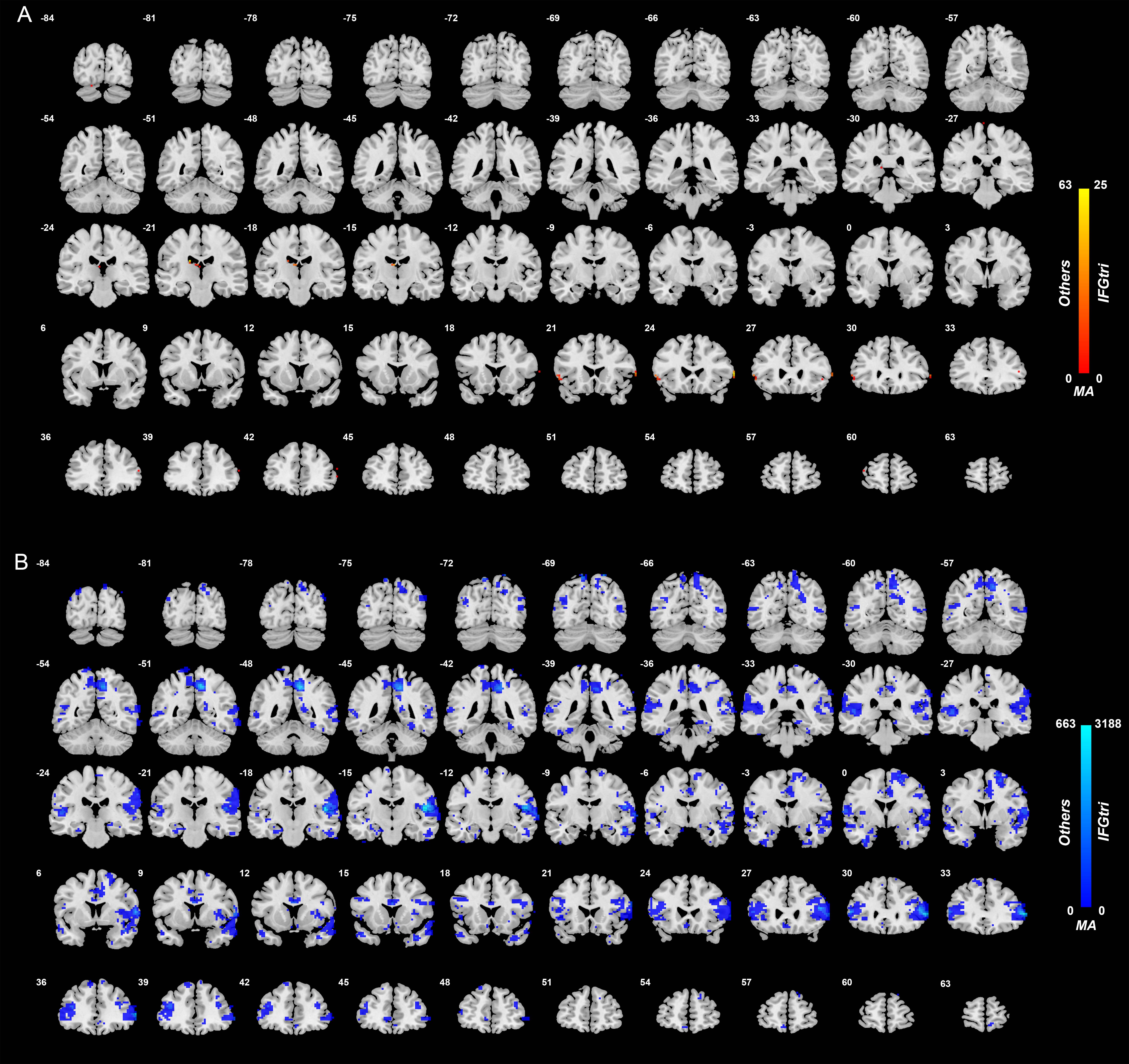


**Figure S7.** Anatomical location of voxels with significantly higher (A) and lower (B) functional connectivity with the inferior frontal gyrus (opercular part) in medicated patients - non-medicated patients obtained from the voxel-based Association Study. Blue indicates voxels with lower functional connectivity in medicated depressed patients, and red/yellow indicates voxels with higher functional connectivity in medicated depressed patients.


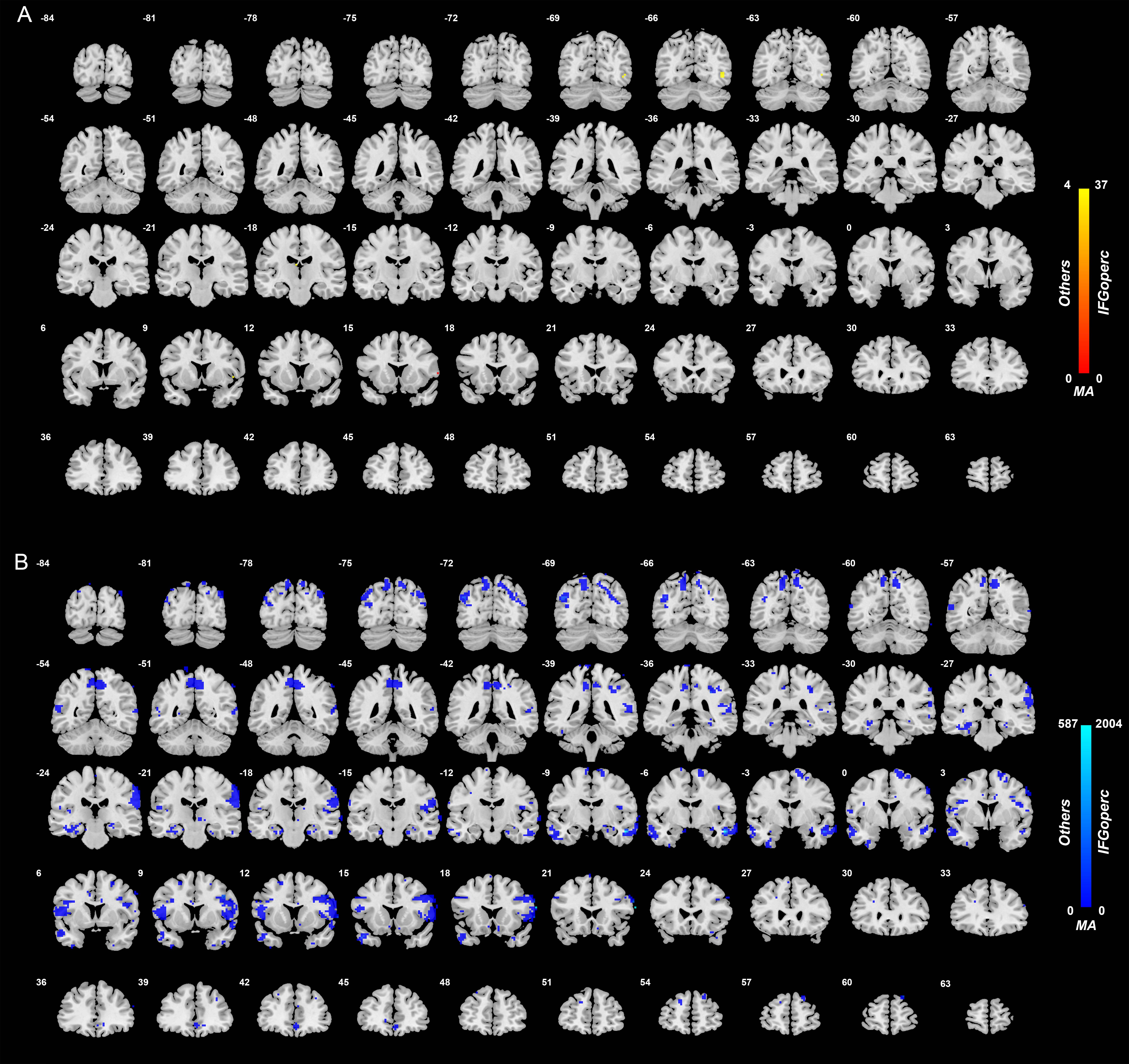


**Figure S8. Comparison of differences in functional connectivity for the right (A) vs the left (B) inferior frontal gyrus in depression.** Anatomical location of voxels with significantly higher functional connectivity with the inferior frontal gyrus (both triangular and opercular parts in non-medicated depression (patients - controls) obtained from the voxel-based Association Study. Red/yellow indicates voxels with higher functional connectivity in patients.


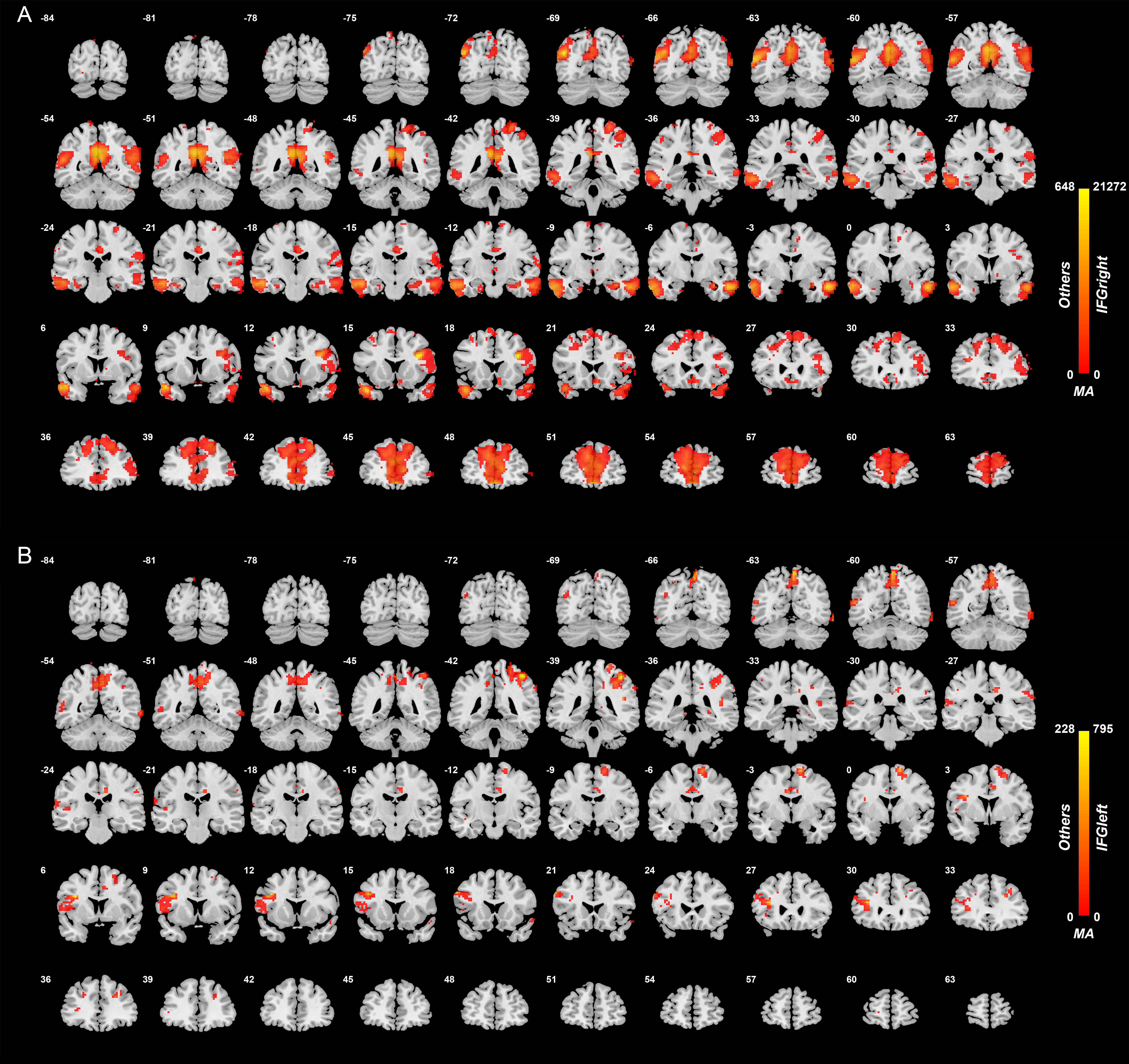


**Figure S9. Functional connectivity difference for 157 medicated patients - 254 controls.** B: Blue indicates voxels with lower functional connectivity in medicated depressed patients. A. Red/yellow indicates voxels with higher functional connectivity in medicated depressed patients. Voxels are shown that are significant at significantly different at p<10^-4^. Voxels are included with different functional connectivities involving the medial and lateral orbitofrontal cortex and the inferior frontal gyrus pars triangularis and pas opercularis.


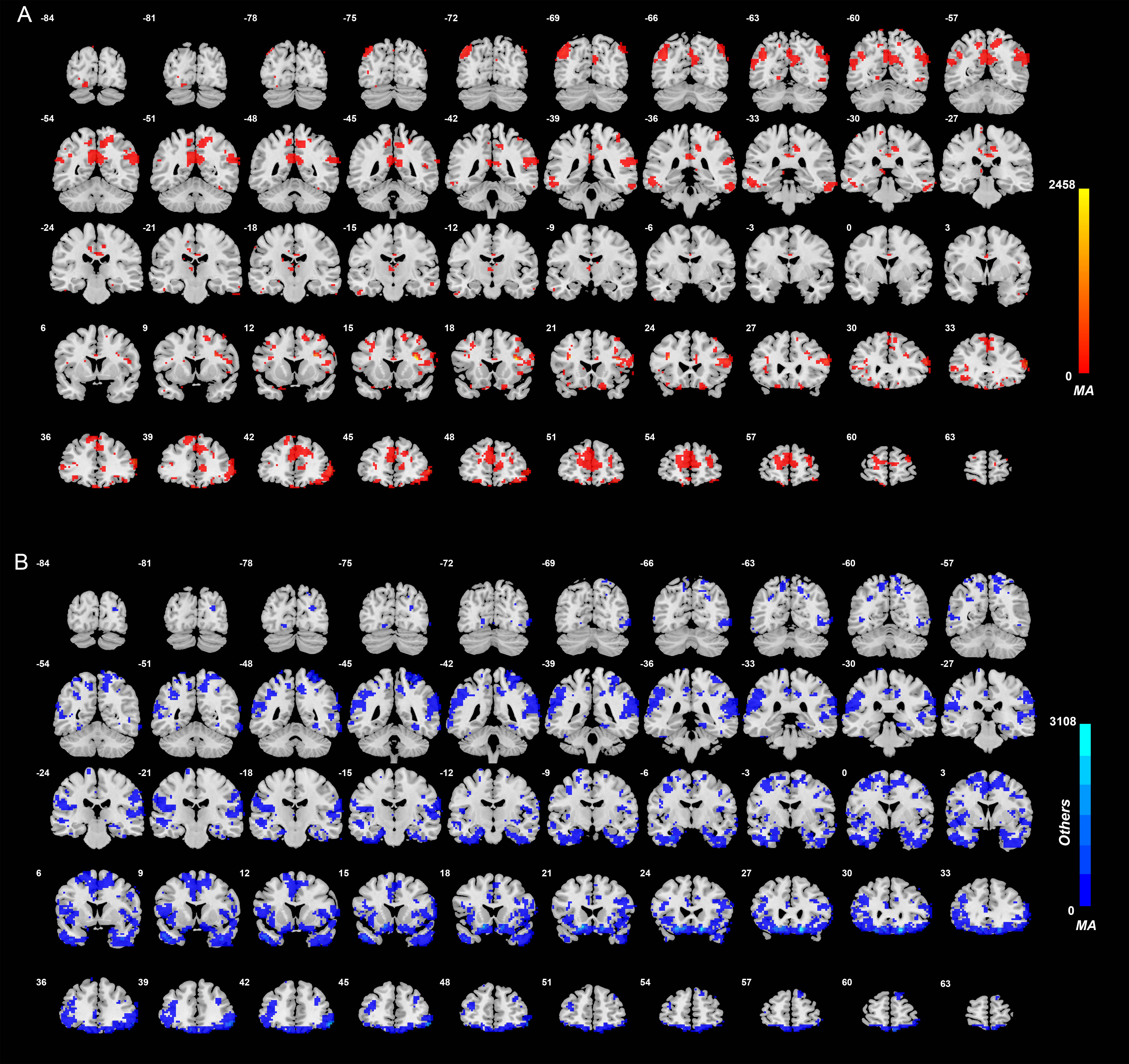


References

Beck, A. T. & Beamesderfer, A. (1974) 'Assessment of depression: the depression inventory', *Modern Problems of Pharmacopsychiatry*, **7**(0), pp. 151-169.

Cheng, W., Rolls, E. T., Qiu, J., Liu, W., Tang, Y., Huang, C. C., Wang, X., Zhang, J., Lin, W., Zheng, L., Pu, J., Tsai, S. J., Yang, A. C., Lin, C. P., Wang, F., Xie, P. & Feng, J. (2016) 'Medial reward and lateral non-reward orbitofrontal cortex circuits change in opposite directions in depression', *Brain*, **139**(Pt 12), pp. 3296-3309.

Hamilton, M. (1960) 'A rating scale for depression', *Journal of Neurology, Neurosurgery and Psychiatry*, **23**, pp. 56-62.

Rolls, E. T., Joliot, M. & Tzourio-Mazoyer, N. (2015) 'Implementation of a new parcellation of the orbitofrontal cortex in the automated anatomical labeling atlas', *Neuroimage*, **122**, pp. 1-5.
